# Faecal Microbiome Transplantation as a Solution to Chronic Enteropathies in Dogs: A Case Study of Beneficial Microbial Evolution

**DOI:** 10.3390/ani11051433

**Published:** 2021-05-17

**Authors:** Michele Berlanda, Giada Innocente, Barbara Simionati, Barbara Di Camillo, Sonia Facchin, Maria Cecilia Giron, Edoardo Savarino, Federico Sebastiani, Francesca Fiorio, Ilaria Patuzzi

**Affiliations:** 1 Department of Animal Medicine, Production and Health, University of Padova, 35020 Padova, Italy; michele.berlanda@unipd.it; 2 Research & Development Division, EuBiome S.r.l., 35129 Padova, Italy; giada.innocente@eubiome.it (G.I.); direzione@eubiome.it (B.S.); barbara.dicamillo@unipd.it (B.D.C.); sonia.facchin@unipd.it (S.F.); cecilia.giron@unipd.it (M.C.G.); edoardo.savarino@unipd.it (E.S.); federico.sebastiani@eubiome.it (F.S.); 3 Department of Information Engineering, University of Padova, 35131 Padova, Italy; 4 Department of Comparative Biomedicine and Food Science, University of Padova, 35020 Padova, Italy; 5 Department of Surgery, Oncological and Gastrointestinal Science, University of Padova, 35121 Padova, Italy; 6 Pharmacology Building, Department of Pharmaceutical and Pharmacological Sciences, University of Padova, 35131 Padova, Italy; 7 Clinica Veterinaria Airone, 36015 Vicenza, Italy; info@clinicaveterinariaairone.it

**Keywords:** FMT, microbiome, dogs, faecal microbiome transplantation, chronic diseases, enteropathy, NGS, 16 rRNA gene

## Abstract

**Simple Summary:**

Chronic enteropathies are common gastrointestinal diseases in domestic dogs characterised by long-term duration, often impairing quality of life both for pets and owners. It has been demonstrated that the gut microbial community plays a central role in defining the host health status. Indeed, among a variety of biological functions, gut microbiota are involved in the metabolism of nutrients, in training the immune system and in preventing the gastrointestinal ecosystem from being colonised by pathogens. In chronic intestinal diseases, the equilibrium of the gut microbial population is largely impaired, as a consequence of both disease and therapy (e.g., antibiotic treatment). Faecal microbiota transplantation has the aim to restore a balanced microbial population in the patient by simply implanting a healthy gut microbiota derived from a healthy donor to a diseased animal. In doing so, the eubiotic community—and the extensive network of beneficial cross-feeding interactions—are transferred to the receiver’s gut as a whole, favouring the patient to renew a healthy intestinal ecosystem. In this work, we report the encouraging results of a faecal transplantation on a 9-year-old dog suffering from chronic enteropathy for the last 3 years. After the treatment, the dog’s appetite, body weight and vitality were restored, with complete disappearance of gastrointestinal and systemic symptoms.

**Abstract:**

Chronic enteropathies (CE) are gastrointestinal diseases that afflict about one in five dogs in Europe. Conventional therapeutic approaches include dietary intervention, pharmacological treatment and probiotic supplements. The patient response can be highly variable and the interventions are often not resolutive. Moreover, the therapeutic strategy is usually planned (and gradually corrected) based on the patient’s response to empirical treatment, with few indirect gut health indicators useful to drive clinicians’ decisions. The ever-diminishing cost of high-throughput sequencing (HTS) allows clinicians to directly follow and characterise the evolution of the whole gut microbial community in order to highlight possible weaknesses. In this framework, faecal microbiome transplantation (FMT) is emerging as a feasible solution to CE, based on the implant of a balanced, eubiotic microbial community from a healthy donor to a dysbiotic patient. In this study, we report the promising results of FMT carried out in a 9-year-old dog suffering from CE for the last 3 years. The patient underwent a two-cycle oral treatment of FMT and the microbiota evolution was monitored by 16S rRNA gene sequencing both prior to FMT and after the two administrations. We evaluated the variation of microbial composition by calculating three different alpha diversity indices and compared the patient and donor data to a healthy control population of 94 dogs. After FMT, the patient’s microbiome and clinical parameters gradually shifted to values similar to those observed in healthy dogs. Symptoms disappeared during a follow-up period of six months after the second FMT. We believe that this study opens the door for potential applications of FMT in clinical veterinary practice and highlights the need to improve our knowledge on this relevant topic.

## 1. Introduction

Chronic enteropathies (CE) are a frequent and frustrating cause of illness in dogs [1], defined as persistence of gastrointestinal symptoms such as vomiting and diarrhoea for three or more weeks. In humans and in dogs, both chronic and acute enteropathies exhibit imbalanced composition of intestinal microbiota, defined as dysbiosis, which has been recognized as a major player in the development of these diseases [2,3,4,5,6]. Moreover, as previously reported, CE are characterized by decreased faecal bacterial diversity and richness [7] with possible impact on the primary functions of gut microbiota. These functions include protection against pathogenic challenge, maturation of the host’s innate and adaptive immune responses, regulation of the host’s metabolism, and maintenance of the structural integrity of the gut mucosal barrier [8]. In term of microbiome composition, animals with CE exhibit greater intra-individual distance compared to healthy animals [9]. Some studies have shown the existence of differentially abundant microorganisms among dogs with different gastrointestinal diseases, e.g., between acute non-haemorrhagic diarrhoea (NHD), acute haemorrhagic diarrhoea syndrome (AHDS), intestinal bowel disease (IBD) [3] and between food-responsive and non-food-responsive CE [9]. Further studies are needed to establish whether a specific dysbiosis profile can be associated to a specific disease.

In addition to conventional therapeutic options for CE—e.g. specific diet, antimicrobials and immunosuppressive drugs [10]—manipulation of gut microbiota is a more and more frequently-adopted approach. Faecal microbiota transplantation (FMT) is a treatment consisting in the transfer of a faecal infusion from a healthy individual (donor) to a sick individual, with the aim of solving dysbiosis of the recipient gut microbiota [11]. In humans, it is clear that FMT recipients can acquire and maintain the transplanted microbiota and there is no particular doubt about its beneficial effect in patients with *Clostridium difficile* infection [11,12]. In veterinary medicine, the practice of microbiota transplantation has been used for many years, especially in horses [13,14,15] and ruminants [16]. In dogs, however, this practice is still limite—and despite the growth in interest, few studies have reported its application and effectiveness in animals with chronic enteropathies [17,18,19,20,21,22,23,24,25,26,27]. This is probably related to the fact that transplantation has been generally performed by endoscopy or enema, with the obvious limitations related to the availability of the technique and its invasiveness [16]. On the other hand, the oral route is still underutilized, due to the lack of a standardised and available method of FMT administration [17,20,23,27]. To date, no moderate or severe side effects of FMT in dogs have been reported, probably due to the limited data availability [16]. On the other hand, in humans, abdominal pain, worsening in stool consistency, nausea and vomiting have been described as common but mild complications [28]. To our knowledge, infectious complications in humans have been rarely reported [29,30], with only two cases reporting fatal outcomes [31]. It is therefore necessary to adopt specific precautions in the choice of donors, especially to exclude the transmission of multidrug-resistant bacteria [16,32].

This case report documents the clinical course and the gut microbiota evolution in a dog with CE, undergoing FMT by oral administration of a standardised formulation.

## 2. Materials and Methods

### 2.1. Case History

A 9-year-old intact male mixed-breed dog named Bruno was examined in a general practice because of a chronic gastrointestinal disorder. The dog had a three-year history of alternating diarrhoea with defecation difficulties, associated with tenesmus, haematochezia and vomiting at least once a month. During the previous three years the dog had undergone numerous diet changes (homemade, high-fibre and hydrolysed) without achieving substantial improvements. The dog was regularly vaccinated and the heartworm prevention program was in place.

On physical examination, the dog was in fair body condition, weighing 7.5 kg and had a body condition score of 4/9. Clinical exam was unremarkable. During the rectal examination, he seemed to experience mild pain. Further diagnostic tests included complete blood count, serum biochemistry (including serum trypsin-like immunoreactivity (TLI)), folate and cobalamin. Faecal analysis and abdominal ultrasound examinations were performed, as well. Complete blood count revealed mild eosinophilia (1.85 × 10^3^/μL; normal range 0.05–1 × 10^3^/μL). Biochemistry, serum electrophoresis and faecal analyses were unremarkable. Abdominal ultrasound examination was normal except for a generalized corrugation of the intestinal wall and an increased thickness of the colonic mucosa. At the first examination, Canine chronic enteropathy clinical activity index (CCECAI) was 6, indicative of a moderate disease.

The dog was treated with fenbendazole (50 mg/kg PO, q24h) for 5 days and ranitidine (2 mg/kg PO, q12h) for 14 days. A switch to a hydrolysed diet and supplementation with probiotics and psyllium seeds was suggested. Antibiotic therapy with metronidazole and spiramycin (12.5 mg/kg and 75.000 UI/kg, respectively) for 14 days was prescribed following an episode of haemorrhagic diarrhoea the day after the first examination.

Five months later, following a series of episodes of haemorrhagic diarrhoea, steroid therapy with prednisolone (0.6 mg/kg) was started.

Two months later, only a slight improvement in symptoms was noted, and the prednisolone dose was increased (0.9 mg/kg PO, q24h) with reduction of intestinal symptoms, but appearance of polyphagia and increased levels of serum alanine aminotransferase (ALT). The dose of prednisolone was gradually reduced to 0.3 mg/kg, resulting in symptoms relapse. Endoscopy was proposed from the general practitioner and the owner agreed. Flexible gastroduodenoscopy and colonoscopy were performed. The gastric mucosa surface exhibited slight multifocal hyperaemia and moderate diffuse mucosal edema. Duodenal endoscopy showed multifocal areas of hyperaemia and a marked reduction of intestinal villi. Colonoscopy revealed diffuse multifocal hyperaemia and multiple areas of erosion in the ascending colon. Multiple biopsies were performed that showed a low grade follicular chronic gastritis, a medium grade lymphoplasmacytic enteritis and plasmocytic chronic colitis.

Antibiotic therapy with tylosin (10 mg/kg, PO, q12 hr) was started and prednisolone was interrupted. The patient benefited from a reduction in the severity of intestinal symptoms but not in the frequency of clinical manifestations, continuing to show monthly episodes of haematochezia and vomiting nearly twice a month (CCECAI = 4). In the following weeks, vomiting episodes increased in frequency. Then tylosin administration was reduced to once a day and budesonide therapy was proposed. The owner refused budesonide therapy due to the potential side effects.

Due to the poor response to treatment and the refusal of budesonide therapy, FMT was proposed. The owner accepted, on light of the fact that preliminary data report that FMT is a safe procedure. Ongoing therapies were thus suspended.

A timeline figure showing case history, FMT and follow-up is available as Supplementary Figure S1.

### 2.2. Faecal Microbiota Transplantation

The patient underwent FMT twice, 8 months apart. In each transplantation the patient was given 1 capsule a day for 30 days. The capsules were purchased from AnimalBiome Inc. (Oakland, CA, USA) as two different FMT kits of 30 days each and ensured the delivery of concentrated lyophilized stool. As the two kits showed some differences in the taxonomic content, we decided to consider the two profiles as coming from two different donors, that were symbolically labelled Donor_1 and Donor_2.

### 2.3. Control Population

A set of 94 samples coming from V3-V4 16S rDNA sequencing of healthy dogs’ faeces was considered as a control population for comparison with the patient’s results. Data for this population were retrieved from Scarsella et al. [33]. In detail, among all the available samples, only the ones belonging to the baseline condition, i.e., samples collected at the first sampling time point (T0), were considered. Furthermore, samples characterised by a sequencing depth lower than 30,000 reads were discarded to obtain more robust and reliable data. The final list of the selected samples is contained in the Supplementary File_S1. From these data, reference ranges (10th–90th percentile) for taxa proportional abundances were calculated at species (File_S2), genus (File_S3) and family (File_S4) levels.

### 2.4. Microbiota Analysis

Faecal samples from the receiver and the second transplantation capsules (Donor_2) were processed and sequenced by BMR Genomics (Padua, Italy) according to the procedures described in paragraph 2.4.1. Conversely, no material from the first FMT capsules (Donor_1) was available for direct inspection at the start of the present study. Information about the Donor_1 composition was then requested directly from the capsules’ producer, which made available the final taxonomic composition (File_S5). Since raw FASTQ files and bioinformatic analysis details were not shared with the authors of the present work, Donor_1 taxonomic information was consequently used only for high taxonomic level evaluations (i.e., phylum-level), for which the taxonomic assignment is less prone to the well-known biases introduced by the choice of different tools, parameters and reference databases.

#### 2.4.1. Sample Collection and DNA Extraction for Microbiota Analysis

50–100 mg of faecal samples was collected with a swab, diluted in a collection tube (BEAVER Biomedical Engineering, Cat #43903) and stored at room temperature for a maximum of 7 days. Cell lysis was performed, combining chemical and mechanical methods (QIAamp PowerFecal DNA Kit, QIAGEN), starting from 250 μL of diluted faecal sample or 100 mg of FMT preparation. Total DNA was extracted from 200 mL of the lysate, using Cador Pathogen 96 QIAcube HT Kit (QIAGEN), following the manufacturer’s instruction. Total DNA was resuspended in 100 μL of nuclease-free water and stored at −20 °C until preparation for sequencing.

#### 2.4.2. 16S rRNA Gene Amplicon Sequencing

16S rRNA gene was amplified by using a standard protocol and modified primers [34]. Briefly, the PCR conditions were as follows: initial denaturation at 94 °C for 1 min, followed by 25 amplification cycles with denaturation at 94 °C for 30s, annealing at 55 °C for 30 s, and extension at 68 °C for 45 s. At the end of the cycles, an extension step at 68 °C for 7 min was performed. The primers used were specific for the V3–V4 region of the 16S rRNA gene: pro341f: 5′-TCGTCGGCAGCGTCAGATGTGTATAAGAGACAGCCTACGGGNBGCASCAG-3′ and Pro805R: 5′-GTCTCGTGGGCTCGGAGATGTGTATAAGAGACAGGACTACNVGGGTATCTAATCC-3′. Amplicons were purified through magnetic beads Agencourt XP 0.8× (Beckman Coulter, Brea, CA, USA) and amplified through HiSeq by using Index Nextera XT kit (Illumina, San Diego, CA, USA). All amplified sequences were normalized by SequalPrep (Thermo Fisher, Waltham, MA, USA) and precipitated through magnetic beads (Agencourt XP 0.8×). Libraries were sequenced onto MiSeq (Illumina, San Diego, CA, USA) following the V3-300PE strategy. The final FASTQ files obtained from the sequencing procedure are available in Supplementary File_S5.

#### 2.4.3. Bioinformatic Analysis

Forward and reverse reads were pre-processed and assembled using the Quantitative Insights into Microbial Ecology pipeline (QIIME2, version 2020.8) [35]. First, primer sequences removal was performed by means of cutadapt [36] considering no indels, an error rate equal to 0 and an overlap of 10 nucleotides, and allowing wildcard read matching (--p-no-indels; --p-error-rate 0; --p-overlap 10; --p-match-read-wildcards). The reads in which no adapter sequence was found were discarded (--p-discard-untrimmed). Then, the amplicon sequence variant (ASV) table was obtained by means of a de novo clustering procedure using the DADA2 [37] bioinformatic tool plugin. The taxonomic assignment of each ASV was determined using the Greengenes database [38] (version 13_8) and two Naive Bayes classifiers—one for the healthy samples and one for the remaining samples— that were trained on the target region selected for the present study, considering the different primer pair adopted in this study with respect to Scarsella et al. [33]. Taxon names in square brackets were proposed by Greengenes curators but the nomenclature is still contested.

Alpha (Richness, Pielou and Shannon indices) and beta (Bray-Curtis dissimilarity) diversity were calculated for microbial community diversity analysis applying a rarefaction level equal to 17043, i.e., the highest sequencing depth that allowed us to preserve all the samples involved in this study. This cut-off was chosen after verification (by means of a rarefaction plot (Figure S2)) that all the samples presented an adequate sequencing depth and that the chosen threshold was placed after each rarefaction curve had reached its plateau. Additionally, beta diversity measure was used for ordination analysis with PCoA technique. Alpha diversity analysis was performed via QIIME2 dedicated plugins, while beta diversity calculation and ordination plot production were performed in R (version 4.0.2) using phyloseq (version 1.32.0) and vegan (version 2.5-7) packages. For the latter task, data were previously normalized using GMPR tool [39] (version 0.1.3) to allow for robust comparison between samples.

## 3. Results

### 3.1. Clinical Outcomes

In the interval between the two FMT and four months after the second FMT, the dog was examined for the purposes of monitoring its health status not related to intestinal disease (e.g., benign prostatic hyperplasia). During the administration of the FMT, the dog showed a progressive improvement and at the end of the month no longer presented episodes of diarrhoea and only presented occasional episodes of haematochezia and non-alimentary vomiting. CCECAI was 4. Then, a second transplantation was made in order to corroborate the initial benefit. At the last follow-up visit, the patient was in good general condition and had a good appetite. The haematochezia and vomiting disappeared for at least six months and were associated with a body weight gain of 20%. Complete blood count and serum biochemistry were assessed, with results comparable to physiological levels, as confirmed by the CCECAI value of 0. Furthermore, no clinical side effects potentially correlated with FMT, including diarrhoea, vomiting, or abdominal pain, were reported by the owners.

### 3.2. Microbiome Analysis

A total of 689,729 reads were obtained from the sequencing of the 9 analysed samples, with a mean value of 76,636.56 and a standard deviation (SD) of 16,261,74. After filtering, denoising, merging and chimera removal steps, a total of 380,489 reads were retained (mean: 42,276.56; SD: 12,326.98), equivalent to the 55.17% of input reads (mean: 54.45%; SD: 8.54%).

#### 3.2.1. Microbial Composition

**Figure 1:**
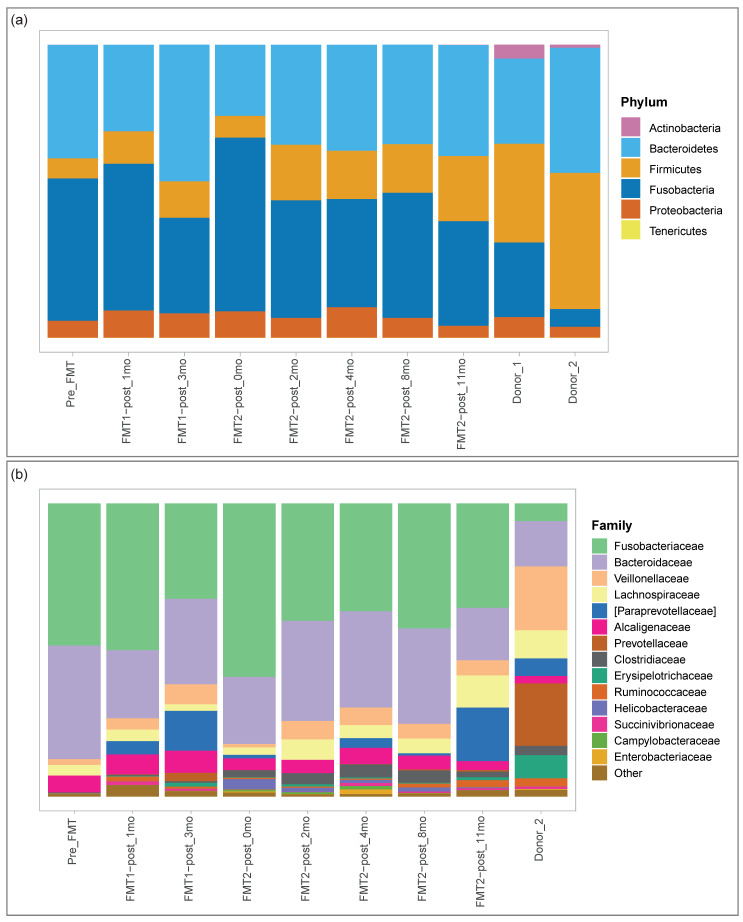
Donors’ and receiver’s faecal microbiome composition. The microbial composition of each sample is represented by means of a stacked bar plot. (**a**) Microbial composition at phylum level; (**b**) Microbial composition at family level. The most proportionally abundant (in mean) families are explicitly shown, while the remaining portion of microbial contributions is grouped in the Other category. Donor_1 information was only included at phylum level (see Methods).

In Figure 1, we report the microbial composition of the receiver at different time-points as well as the profiles of the donors from which the capsules used for the two transplantation procedures were obtained. The complete community composition at species, genus and family levels can be found in the Supplementary Tables (Table S1, Table S2 and Table S3 respectively). As regards the phylum level (Figure 1, panel (a)), *Firmicutes* and *Bacteroidetes* were the prevalent phyla of the donors’ microbiome, while the first stages of the patient’s microbiome were characterised by a high abundance of *Fusobacteria* taking the place of the *Firmicutes* phylum. After the first and second transplantation the receiver’s microbiome showed a shift towards the donors’, with the decrease in percentage of *Fusobacteria* and the corresponding increase of *Firmicutes*. Our case-study lacked statistical analysis to confirm a trend in the microbiome evolution; however, we noted the potential appearance of two patterns of behaviour (Figure 1, panel (b)). *Fusobacteriaceae*, *Bacteroidaceae*, *Prevotellaceae*, [*Paraprevotellaceae*], *Ruminococcaceae*, *Veillonellaceae* and *Erysipelotrichaceae* first shifted towards donor’s percentage after the first FMT. Immediately after the second transplantation, they showed a regression to the initial values and then then resumed the initial trend. Conversely, *Clostridiaceae*, *Lachnospiraceae* and *Alcaligenaceae* remained quite stable after the first transplantation and gradually moved to the donor’s percentages after the second transplantation. The analysis of the control population identified the common core microbiome, here defined as the set of microbial taxa that occur at least in 95% of healthy individuals [40]. The core microbiome included 12 families: *Prevotellaceae*; *Veillonellaceae*; *Turicibacteraceae*; [*Paraprevotellaceae*]; *Bacteroidaceae*; *Peptostreptococcaceae*; *Coriobacteriaceae*; *Fusobacteriaceae*; *Ruminococcaceae*; *Erysipelotrichaceae*; *Clostridiaceae* and *Lachnospiraceae*. While the donor possessed all the core families, the receiver lacked *Prevotellaceae*, [*Paraprevotellaceae*] and *Turicibacteraceae* before the treatment. The first two families were then acquired after the transplantation.

#### 3.2.2. Diversity Analysis

**Figure 2:**
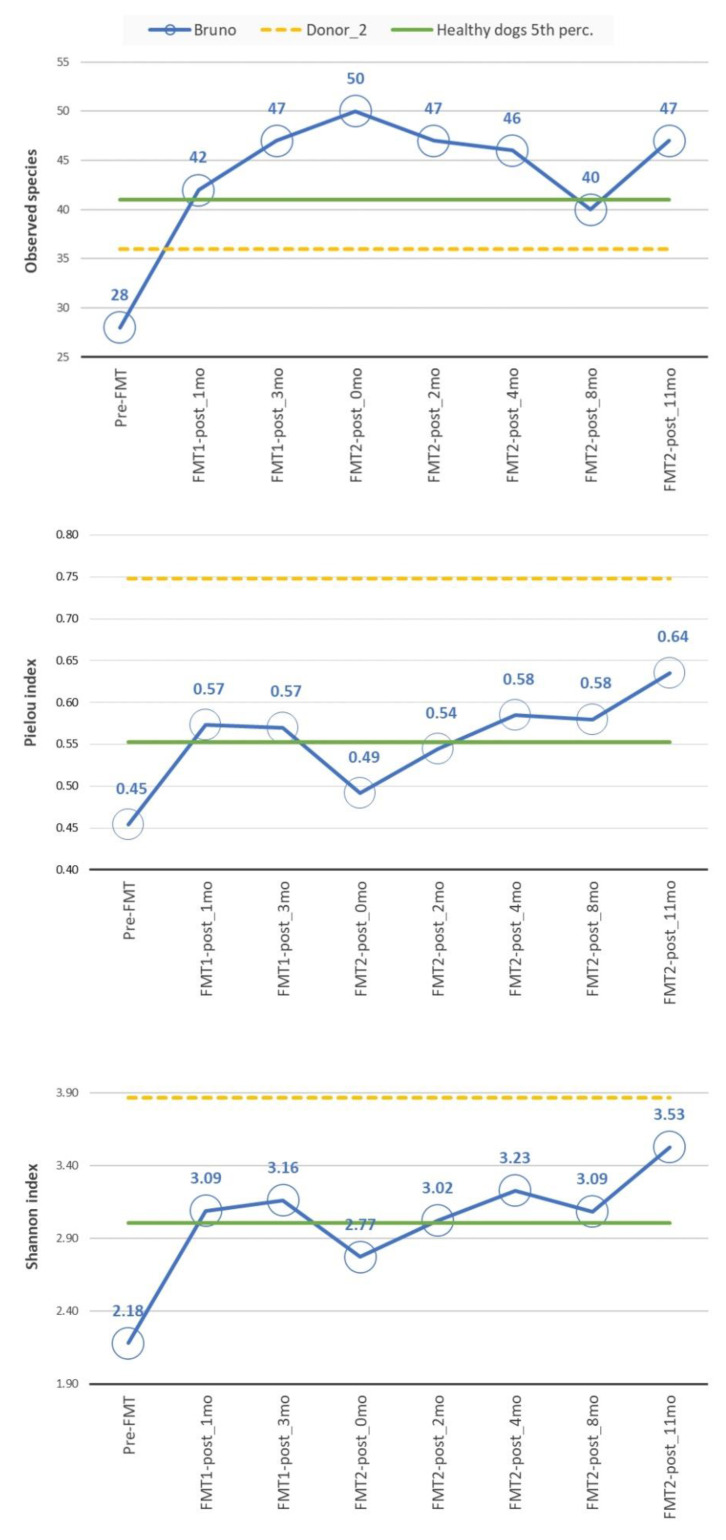
Alpha diversity analysis. The values for species Richness (**top**), Pielou index (**middle**) and Shannon index (**bottom**) are reported as a blue polyline for the patient (Bruno), while the reference values for the FMT capsule and the minimum healthy threshold (5th percentile of healthy samples’ values) are reported in dashed yellow and solid green, respectively.

Figure 2 shows the results of alpha diversity analysis, comparing the patient’s dynamics along sampling time points and the reference values for the FMT capsule and the minimum healthy threshold, chosen as the 5th percentile of healthy samples’ alpha diversity values. The first relevant result was the globally growing trend of all the three indices, showing a general improvement of the gut microbial environment health and a shift of the microbiome condition through the values characterizing both the donor and the healthy control population.

Although they shared a similar global trend, the indices had a different local behaviour. The Richness index (number of observed species) had a continuous growing trend until the fourth time point, stabilizing its value around 46/47 species. This was theoretically compatible with the FMT procedure, in which new species from the donor were introduced in the patient, causing the richness to initially grow. Over time, only the species that underwent the so-called engraftment remained in the patient microbiome, while some species were lost, causing the richness to slightly decrease. A unique drop in richness was registered at the second-to-last sampling time. However, this behaviour could be linked to a reason more technical than biological. Indeed, the “FMT2-post_8mo” was the most problematic sample because it showed the highest percentage of reads discarded during the pre-processing steps, with a larger amount lost in the filtering step. The final amount and quality of retained reads were acceptable for the inclusion of the sample in the study, but it cannot be excluded that the above-mentioned peculiarities may have had an influence on the final metrics.

As regards evenness (Pielou) and diversity (Shannon) indices, they both shared the same time-varying behaviour. After an initial increase due to the first FMT, both the evenness and the diversity saw a slight decrease to a lower value, albeit one that was still higher than the pre-FMT one. After the second FMT, both indices passed the healthy control population threshold and increased at each sampling time point, showing a progressive shift to the donor’s and the control population’s characteristics.

**Figure 3:**
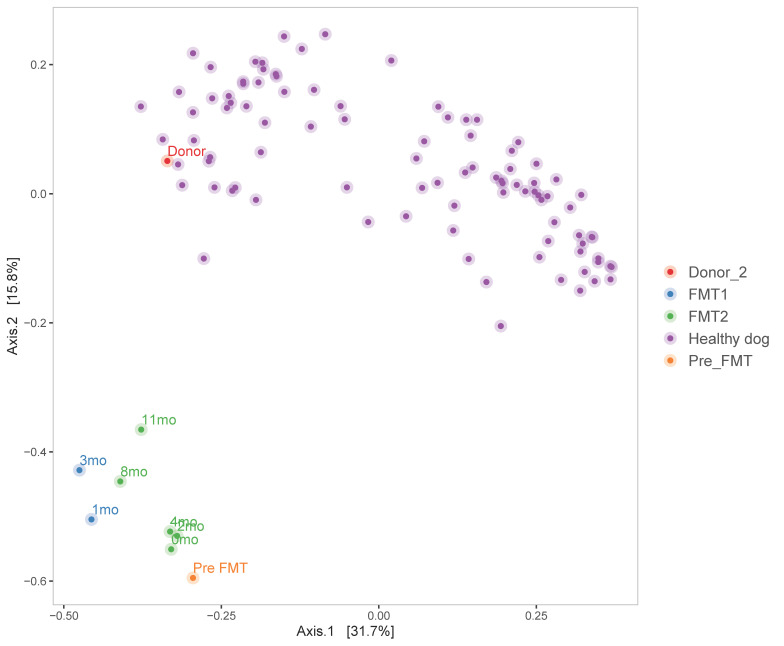
PCoA plot on Bray–Curtis beta diversity measure. The normalized (GMPR) data are represented with different colours to highlight different groups: purple for healthy controls, light blue for samplings after the first FMT, green for samplings after the second FMT, orange and red for pre-FMT and Donor_2 samples, respectively.

The movement towards a healthier condition is also visible in Figure 3, in which a clear path is distinguishable for the patient’s samples. Indeed, the analysis showed that the pre-FMT microbiome profile was the farthest from the healthy/donor’s cluster (File_S6). Moreover, following what alpha diversity results and clinical evaluations already showed, after the first FMT treatment, the microbiome grew more similar to the donor’s (see 1mo and 3mo samples, in light blue in the figure). Data also showed that after 6 months post-transplant the patient had a little regression (0mo sample, in green), but that after the second transplant, the shift towards the healthy cluster started again, to finally reach the shortest distance in the final observed time point (11mo).

## 4. Discussion

The present study describes the case of a dog with chronic enteropathy refractory to conventional treatments, successfully treated with two consecutive 1-month FMT.

FMT in dogs is still in its infancy, and the scientific literature in veterinary practice, unlike the human one, is still quite fragmentary. This unfortunately limits its use by veterinarians as an alternative to standard treatments.

In veterinary medicine, the greatest evidence of efficacy was found in cases of acute diarrhoea [19], acute haemorrhagic diarrhoea syndrome (AHDS) [18] and infectious diarrhoea [25]. Greater caution is still needed in considering the positive clinical results obtained in dogs with chronic enteropathy [20,21,22,24].

In the present case report, the treatment with FMT was found to effectively control symptoms of chronic enteropathy and drastically reduce the CCECAI in the absence of immunosuppressive therapies and/or antibiotic therapy, with an undoubted improvement in the quality of life of the patient and the owner.

In this discussion, even if the changes in microbiome lacked statistical significance and the community shift toward the donor’s profile could not be demonstrated, the potential role of the taxa that showed greater variation merits further consideration in view of the positive clinical outcome.

[*Paraprevotellaceae*] and *Prevotellaceae* were missing in the receiver before the treatments. These two core families are usually depleted in the duodenum of dogs with idiopathic inflammatory bowel disease, along with *Veillonellaceae Lachnospiraceae*, *Ruminococcaceae* and *Erysipelotrichaceae* [41]. In our case study, all these families showed a progressive increase over time. Different studies have clarified the role of these taxa in gut health. It has been proven that *Lachnospiraceae* and *Ruminococcaceae* induce an anti-inflammatory response through the induction of Treg cells [42]. Furthermore, these bacterial groups, along with *Erysipelotrichaceae*, are believed to be the major producers of short chain fatty acids (SCFA), the energy source of colonocytes. Dogs with CE usually have an altered faecal SCFA concentration accompanied by significant changes of the faecal microbiota [2,43].

*Clostridiaceae* showed a marked rise after the second FMT. This heterogeneous core family comprises pathogens such as *Clostridium difficile* and common commensals associated with protein digestion [2]. *Clostridium perfringens* and *Clostridium hiranonis* are the main species responsible for the increase of this family (Table S1). Although the former species is thought to be pathogenic in humans, its role in the intestinal health of the dog is complex and still debated. A recent study highlighted that, while omnivores produce SCFA mainly from vegetal fibre fermentation, carnivores possess a metabolic pathway which allows the production of butyrate (a SCFA) from proteins, specifically via butyrate kinase genes coming from *C. perfringens* [44]. In addition to the functional role of this species, some strains possessing *netF* toxin gene seem to cause AHDS [45]. *C. hiranonis*, on the contrary, has an undoubtedly positive role in dog health, since it is one of the main regulators in bile acids (BA) metabolism through the conversion of primary BA in secondary BA [2,9]. This pathway is essential for lipid digestion and regulation of intestinal inflammation, and is commonly altered in chronic gastrointestinal diseases [46,47,48]. Furthermore, secondary BA participate in gut mucosal defences through their antibacterial properties [49,50]. *C. hiranonis* is then a candidate keystone species, since its acquisition, even at low levels, may have a profound impact on the ecological community [2,9].

*Fusobacteriaceae* and *Alcaligenaceae* are normally present in healthy dogs [19,51]. At the same time, dogs with AHDS show increases in *Fusobacteriaceae* and in the genus *Sutterella* (family Alcaligenaceae) [3]. In the present study, these taxa exceeded the 90th percentile of healthy samples’ values before the treatments, and they approached the maximum healthy threshold throughout the study (Table S2, File_S3).

*Helicobacteraceae*, *Succinivibrionaceae*, *Campylobacteraceae* and *Enterobacteriaceae* were present at low abundance in some samples, but their value declined toward zero after the second FMT. These families belong to *Proteobacteria*, a hallmark of dysbiosis usually overrepresented in faecal samples of dogs with chronic enteropathies [43,49]. Their decrease throughout the study—as well as the overall changes in the microbial community—suggests a successful recovery of the gut microbiome, even if our observations were not supported by statistical analysis.

A previous study on the efficacy of FMT in the treatment of canine IBD reported a case in which *Fusobacteria* and *Bacteroidetes* were substantially absent, and *Proteobacteria* predominated before FMT [22]. Repeated FMT treatments successfully resolved the symptoms and the shift of microbiome profile through the donor’s was associated with the clinical outcome.

In our work, the microbiome before the FMT showed an excess of *Fusobacteria* and a substantial deficiency of *Firmicutes* compared to the healthy population. With marked differences from the previous study, we confirmed the efficacy of repeated treatments in case of relapsed CE [22]. The different community dynamics between the two studies can be explained by the evidence of great diversity in microbiome composition in animals with CE [9].

In addition to its effectiveness, the strength of the FMT relied on its excellent safety profile, with a very low number of FMT-related adverse events reported in human and veterinary medicine. No FMT-related side effects were observed in the present case report and only one case of transient worsening of diarrhoea in the 48 h after the FMT has been reported in veterinary literature [20].

FMT is frequently administered to human patients using colonoscopy and oral capsules. Conversely, in studies performed in dogs, rectal enema is the most used route of administration of faecal microbiota [18,21,22,24,25,27]. Other studies reported the use of descending endoscopy [20], the oral route [19,20,23,27], colonoscopy [26] and the orogastric probe [17]. However, the effectiveness of different administration techniques for FMT in dogs has not yet been adequately studied. Some results [20,27] seemed to suggest that the oral route was more effective, perhaps due to bias of the different frequency of administration. The rationale behind the use of the oral route of FMT in the present case report was, then, the simplicity of oral FMT, which allowed serial administration for prolonged periods. Also, the use of a standardised formulation of FMT was helpful in making this procedure so effective.

Regarding patient monitoring, the studies in the literature described very different monitoring protocols. The use of clinical methods such as the CCECAI and the evaluation of stool consistency through validated scales seemed to be the most advisable tools, able to more objectively monitor the response to treatments. However, the availability of HTS techniques for monitoring changes in the intestinal microbiota of patients undergoing FMT offered the opportunity to gather information relevant for clinical implications, e.g., understanding what happens to gut microbiota following FMT. In particular, the time-course monitoring of the two-step treatment allowed us to observe a slight worsening of microbiome-linked metrics (alpha and beta diversity) some months after the first cycle, suggesting that, in some cases, a repeated treatment should be performed to fully recover gut microbiome ideal conditions. Moreover, the identification of specific gut microbiota able to restore a patient to healthy condition could be useful to identify a more precise approach for future studies.

## 5. Conclusions

The present time-varying study supports the clinical efficacy of microbiome transplantation on a chronic enteritis patient, but also highlights some still open questions, e.g., defining indications, methodology and administration protocols for the use of FMT. In particular, optimal duration and number of treatments still remain unsolved points, even for human microbiome transplantation, where available data far exceed that available for pets. With the aim of acquiring more microbiota data to test for optimal treatment parameters, we created a citizen science project called “Pet FMT Project” (www.progettopetfmt.it, accessed on 4 May 2021). The final objective will be the collection of data sufficient enough to perform deep artificial intelligence analyses to fill the above-mentioned gaps and to profile patients and donors, in order to finally match each pathologic profile to its optimal curing donor. This would permit clinicians to further unleash the potential of FMT—and to efficiently move in the direction of cost-effective precision medicines for pets.

## Data Availability

Healthy control population data are available in the related publication at https://doi.org/10.1371/journal.pone.0237874 (accessed on 28 December 2020). Data presented in this study are available in Supplementary File_S5 and have been deposited in the NCBI Sequence Read Archive database under accession number PRJNA722739.

## Supplementary Materials

The following are available online at https://zenodo.org/record/4719155#.YKI2_6ERWUl: File_S1: Healthy samples included in the control population; File_S2: Species level ranges for proportional abundances in healthy population; File_S3: Genus level ranges for proportional abundances in healthy population; File_S4: Family level ranges for proportional abundances in healthy population; File_S5: Raw FASTQ files; File_S6: Bray-Curtis distances from donor and healthy samples’ centroid; Table S1: Species Table; Table S2: Genus Table; Table S3: Family Table; Table S4: Donor_1 composition table; Figure S1: Case report timeline; Figure S2: Rarefaction plot.

## Abbreviations

HTS: high-throughput sequencing; CE: chronic enteropathies; FMT: faecal microbiome transplantation; CCECAI: Canine chronic enteropathy clinical activity index; NHD: Non-Hemorrhagic Diarrhea; AHDS: Acute Hemorrhagic Diarrhea Syndrome; IBD: intestinal bowel disease; SCFA: short chain fatty acids; BA: bile acids.
